# Bifunctional recombinant fusion proteins for rapid detection of antibodies to both HIV-1 and HIV-2 in whole blood

**DOI:** 10.1186/1472-6750-6-39

**Published:** 2006-09-22

**Authors:** Amita Gupta, Vijay K Chaudhary

**Affiliations:** 1 Department of Biochemistry, University of Delhi South Campus, Benito Juarez Road, New Delhi-110 021, India

## Abstract

**Background:**

Availability of accurate diagnostic tests has been helpful in curtailing the spread of HIV infection. Among these, simple, point of care, inexpensive tests which require only a drop of blood from finger-prick and give reliable results within minutes are a must for expansion of testing services and for reaching mobile and marginalised populations. Such tests will not only be a boon for the infrastructure-starved developing and underdeveloped countries but will also be extremely useful in developed countries where post-testing compliance is a major problem. Our laboratory has been involved in developing reagents for heamagglutination-based rapid detection of antibodies to HIV in whole blood using recombinant molecules specific for either HIV-1 or HIV-2. Since it is not required of a screening test to differentially detect HIV and HIV-2, it would useful to create a single molecule capable of simultaneous detection of both HIV-1 and HIV-2 in a drop of blood.

**Results:**

The present paper describes designing, high-level expression and large-scale purification of new molecules comprising recombinant anti-RBC Fab fused to immunodominant regions of envelope sequences from both gp41 of HIV-1 and gp36 of HIV-2. These immunodominant regions of HIV envelope contain cysteine residues, which make disulfide bond and can interfere with the assembly of light chain and heavy chain fragment to make Fab molecule *in vitro*. To circumvent this problem, a series of fusion proteins having different combinations of native and mutant envelope sequences were constructed, purified and evaluated for their efficacy in detecting antibodies to HIV-1 and HIV-2. A chimeric molecule comprising native envelope sequence of gp41 of HIV-1 and modified envelope sequence of gp36 of HIV-2 gave good production yield and also detected both HIV-1 and HIV-2 samples with high sensitivity and specificity.

**Conclusion:**

The new bifunctional antibody fusion protein identified in this study detects both HIV-1 and HIV-2 infected samples efficiently and can be used in place of molecules that detect only HIV-1 or HIV-2. This will make reagent production more economical as only one molecule has to be produced in place of two molecules. Also, it will simplify the testing procedure allowing detection of both HIV-1 and HIV-2 infections in a single drop of blood.

## Background

The AIDS pandemic has already resulted in the death of approximately 21.8 million people worldwide [1] and this number will continue to increase at an alarming rate until corrective measures are taken. Although the major route of transmission is sexual contact, use of contaminated blood and blood products is estimated to have resulted in about 10% of all HIV infections worldwide. Availability of accurate diagnostic tests can certainly help in curtailing spread due to blood products. There has been an explosion in diagnostic technologies in recent times and simple, rapid, inexpensive tests, which require no instrument and can be performed in any remote area preferably with a drop of blood are the need of the hour. Such type of tests will not only be a boon for the developing and under-developed countries where infrastructure is poor and resources are limited, but will also be extremely helpful in developed countries in clinical settings such as emergency rooms where obtaining immediate results could be beneficial. In current practice, it takes between 24 hours to two weeks to get the test results because the testing is conducted primarily in batches at centralized testing laboratories. Delay in availability of test report results in poor compliance in collection of the report and post-test counseling; consequently large number of HIV positive persons unaware of their HIV status, move around in the society [2]. Rapid detection can significantly decrease HIV transmission if these individuals are informed and counseled immediately [3]. Rapid tests will also be useful in immediate identification of pregnant mothers at risk of having HIV infection who could be given antiviral therapy during labor to reduce the incidence of HIV transmission to newborn.

Our laboratory has been involved in development of highly sensitive and specific reagents for detection of antibodies to HIV in blood of infected individuals [4]. These reagents consist of recombinant, monovalent, Fab-based bifunctional antibody molecules having capacity to bind to human RBC on one end and to anti-HIV antibodies (which are present in the blood of HIV infected individuals and are bivalent) on the other end. The reaction leads to cross linking of RBC in the blood of an HIV infected person upon addition of the above-mentioned bifunctional molecule. The cross-linking of RBC is seen as clumping or agglutination of RBCs by naked eyes. On the other hand, there is no cross-linking, if blood from a normal individual is used. The high level production of these antibody fragments and their derivatives involves cytosolic expression as inclusion bodies followed by denaturation and *in vitro *renaturation procedure [5].

Earlier, we have described reagents consisting of Fab fusion proteins, each carrying a single antigen derived either from HIV-1 or from HIV-2, for the differential detection of antibodies to HIV-1 and HIV-2 in a haemagglutination based assay [6]. However, for screening purposes it is not important to differentially detect HIV-1 and HIV-2 antibodies in a sample. Therefore, a single reagent capable of detecting both anti HIV-1 and anti HIV-2 antibodies simultaneously would simplify the testing process and make it a one step test. This can be achieved by mixing HIV-1 specific reagent and HIV-2 specific reagent before adding to the test blood (our unpublished result). However, Fab fusion proteins carrying both antigens on the same Fab molecule would be a better solution as it will reduce the number of molecules required to be purified, thereby simplifying the process of reagent production and usage.

The development of a single molecule containing both HIV-1 and HIV-2 specific immunodominant regions is not straightforward. Both env1 and env2 sequences of HIV contain disulfide bonds, which might interfere with the *in vitro *assembly of LC and Fd chains to form Fab as well as with correct disulfide bond formation within env1 and env2. This can be avoided by using a derivative of envelope antigens carrying serine residues in place of cysteine residues. However, absence of the disulfide bond in the antigen may cause alteration in its reactivity with antibodies present in serum samples from HIV-infected patients [7]. In the present paper, we describe designing, high level expression in *E. coli*, and purification of anti-RBC Fab fusion proteins containing immunodominant regions of both HIV-1 gp41 and HIV-2 gp36, and use of these proteins for successful detection of both HIV-1 and HIV-2 infected individuals, with high sensitivity and specificity.

## Results

### Vector for expression of various proteins

**Figure 1:**
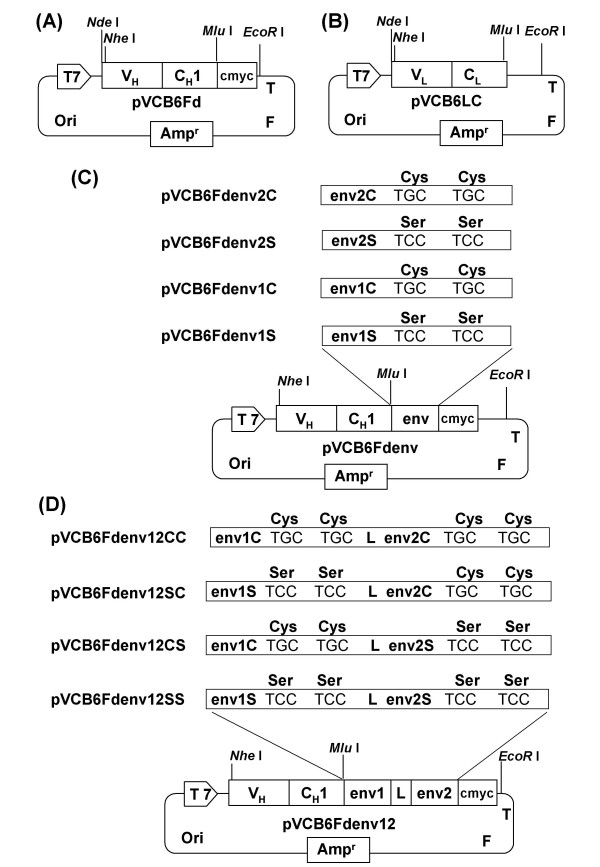
Diagrammatic representation of various plasmids encoding LC, Fd, and Fd fusion proteins. All the plasmids carry insert between *Nhe *I (GCTAGC) and *Mlu *I (ACGCGT) restriction sites and are under the control of T7 promoter. ATG of *Nde *I site (CATATG) is the initiation codon. Only relevant genes and restriction sites are shown. The map is not to the scale. T7, T7 promoter; env1C, 31 amino acids (590–620) of HIV-1 envelope gp41 containing native cysteine residues; env1S, 31 amino acids (590–620) of HIV-1 envelope gp41 with cysteine 605 and 611 replaced by serine residues; env2C, 31 amino acids (581–611) of HIV-2 envelope gp36 containing native cysteine residues; env2S, 31 amino acids (581–611) of HIV-2 envelope gp36 with cysteine 597 and 603 replaced by serine residues; cmyc, decapeptide recognized by monoclonal antibody, 9E10; T, T7 transcription terminator; F+, phage M13 origin of replication; Amp^r^, β-lactamase gene; Ori, ColE1 origin of replication; V_H_, variable domain of the heavy chain of MAb B6; C_H_1, the first constant domain of heavy chain of B6; V_L_, variable domain of the light chain of B6; C_L_, constant domain of light chain of B6. A. Expression vector for B6Fd. B. Expression vector for B6LC. C. Segment of expression vector pVCB6Fdenv showing the cassette between B6Fd and cmyc. The cysteine residues in env1/env2, which make intra-molecular disulfide bond, are shown. The serine residues that replace the cysteine residues in env are shown. D. Segment of expression vector pVCB6Fdenv12 showing the cassette of chimeric env1 and env2 sequence between B6Fd and cmyc. The serine and cysteine residues in env sequences are shown. L, linker.

**Figure 2:**
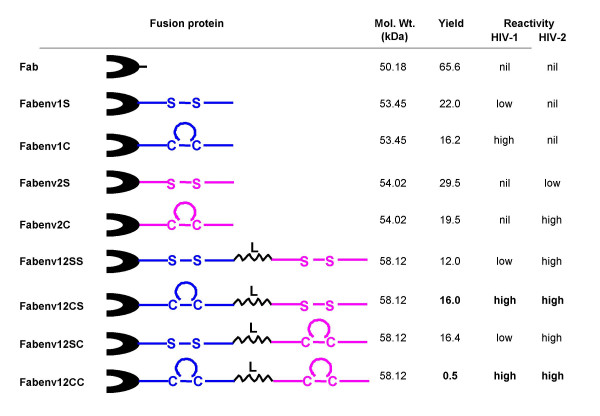
Diagrammatic representation of various Fab molecules along with their molecular weight in kDa. S, serine; C, cysteine; L, spacer with sequence SGGGSGGGASG; yield corresponds to calculated amount of Fab (in mg) obtained from four litre of renatured material (containing 400 mg of total protein). Reactivity of proteins to HIV-1 and HIV-2 serum samples based on Table 2.

pVCB6Fd (Fig. 1A) carries sequence encoding amino acids 1–219 of the heavy chain (Fd; consisting of V_H _and C_H_1) of an anti-RBC monoclonal antibody, B6, as *Nhe *I-*Mlu *I insert. *Nhe *I site in the vector is preceded by *Nde *I site (CATATG) whose ATG constitutes the initiation codon. *Mlu *I site that is used for cloning various envelope sequences is followed by sequence for a ten amino acid tag, cmyc that is recognised by MAb, 9E10 (Fig. 1A). pVCB6LC1140 is similar to pVCB6Fd and carries sequence encoding the light chain (amino acids 1–219) of B6 as *Nhe *I-*Mlu *I insert (Fig. 1B). This plasmid contains a stop codon after the coding sequence of B6LC and preceding *Mlu *I site. In pVCB6Fdenv1C, following Fd, a sequence encoding 31 amino acids (590–620) of HIV-1 gp41 (env1C) has been inserted (Fig. 1C; Table 1). This plasmid expresses a fusion protein B6Fdenv1C (Fig. 2). In pVCB6Fdenv2C, following Fd, a sequence encoding 31 amino acids (581–611) of HIV-2 gp36 (env2C) has been inserted (Fig. 1C; Table 1). This plasmid expresses a fusion protein B6Fdenv2C (Fig. 2). pVCB6Fdenv1S is similar to pVCB6Fdenv1C but expresses a fusion protein, B6Fdenv1S, with cysteine 605 and 611 residues present in native gp41 replaced by serine residues. pVCB6Fdenv2S is similar to pVCB6Fdenv2C but expresses a fusion protein, B6Fdenv2S (Fig. 2), with cysteine 597 and 603 residues present in native gp36 replaced by serine residues.

pVCB6Fdenv12SS (Fig. 1D) expresses a fusion protein B6Fdenv12SS containing Fd of B6 antibody fused at its C-terminus to sequences encoding amino acids 590 to 620 of gp41 with cysteine 605 and cysteine 611 replaced by serine residues, an eleven amino acid linker (SGGGSGGGASG), amino acids 581 to 611 of gp36 with cysteine 597 and cysteine 603 replaced by serine residues and a decapeptide tag, cmyc (Table 1). pVCB6Fdenv12CS, pVCB6Fdenv12SC and pVCB6Fdenv12CC (Fig. 1D), express fusion protein similar to B6Fdenv12SS with cysteine in env1 (B6Fdenv12CS) or env2 (B6Fdenv12SC) or both (B6Fdenv12CC) (Fig. 2, Table 1).

### Expression of various proteins and preparation of inclusion bodies

BL21(λDE3) cells transformed with various plasmids were grown and expression of the desired protein was induced with IPTG. Cells from induced cultures were analysed for expression by SDS-PAGE under reducing conditions. The expressed proteins corresponded to the calculated molecular weight of the respective proteins. Large-scale expression for all the constructs was done at 30°C. The expressed proteins constituted ~15–30% of total cellular protein and the inclusion bodies purified from this cell pellet constituted ~70–80% of total cellular protein (data not shown).

### Assembly of Fab molecules

For the assembly of various B6Fab derivatives, a protocol comprising of *in vitro *denaturation of Fd/Fdenv and LC, and their subsequent renaturation and association was optimised. B6Fd derivatives and B6LC were completely denatured and reduced and then mixed in equimolar ratio. The assembly of Fab was initiated by diluting the mixed Fd and LC in a buffer containing oxidised glutathione. This created proper redox conditions for the refolding of Fd and LC, formation of intramolecular disulfide bonds, association of Fd with LC, and formation of intermolecular disulfide bond to form Fab. The renatured material was dialysed to remove denaturants. This was followed by a series of column chromatography steps to purify monomeric Fab derivatives.

### Purification of various B6Fabenv fusion proteins

A four-step protocol consisting of cation exchange, hydrophobic, anion exchange and gel filtration chromatography was developed to purify various fusion proteins. Fractions obtained at various chromatography steps were analysed for level of purity (by SDS-PAGE) and, for presence of functional and monomeric Fab (by agglutination assay). Fab molecules are monovalent in nature and therefore, can bind to RBCs but cannot cross-link RBCs to give visible agglutination. However, these Fab-coated RBCs can be crosslinked by MAb 9E10, which binds to c-myc tag present at the C-terminus of all Fab molecules. Therefore, all samples containing functional Fab molecules will show agglutination of RBCs on addition of 9E10 and the extent of agglutination will be directly proportional to the amount of active, monomeric Fab in that sample. However, if a sample shows agglutination before addition of 9E10, it indicates that the Fab molecules in that sample are not monomeric but have aggregated to become multimeric. Such samples are not processed further. Purification of B6Fab, B6Fabenv1C and B6Fabenv2C has been described previously [6]. B6Fabenv1S and B6Fabenv2S were purified following protocols similar to those used for B6Fabenv13C and B6Fabenv24C, respectively. The yields obtained for B6Fabenv proteins are shown in Fig. 2. For purification of all the B6Fab fusion proteins containing sequences of both env1 and env2 a modified protocol was developed. The following section describes purification of B6Fabenv12CS in detail.

**Figure 3:**
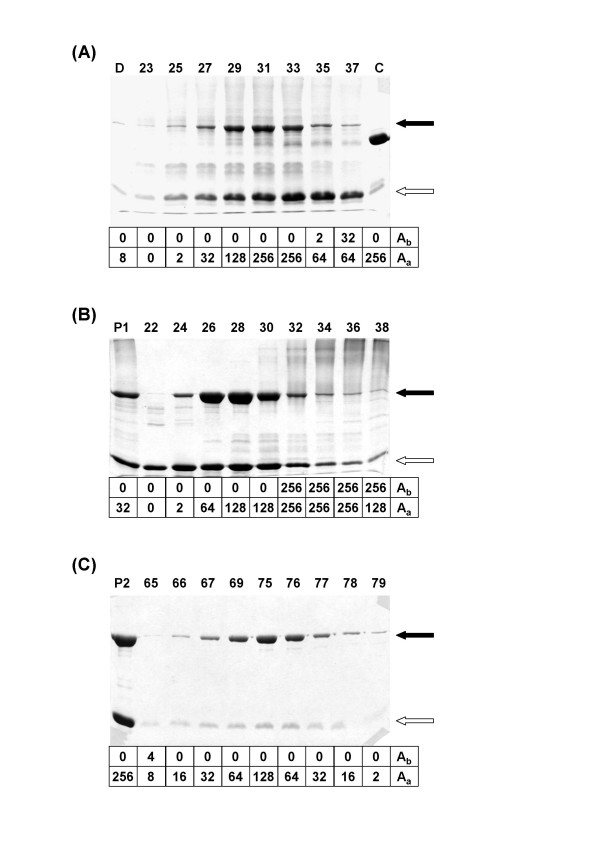
Purification of B6Fabenv12CS. Renatured material (2.3 litre) was subjected to a series of chromatographic purification columns as described in methods. The fractions obtained through each purification step were analysed by electrophoresis on 0.1% SDS-10% PAG under non-reducing conditions. Each lane contains sample equivalent to 10 μl of each fraction. The protein bands were visualised with Coomassie blue R-250 staining. The figure shows Coomassie blue stained gel of the eluted fractions from (A) SP-Sepharose, (B) Q-Sepharose, and (C) Sephacryl S-200 columns. Panel (A): Lane D, dialysed renatured material; Lane 23–37, fractions 23 to 37 of SP-Sepharose column; Lane C, Purified B6Fab protein (control); Panel (B): Lane P1, pool of fractions from SP-Sepharose column; Lane 22–38, fractions 22 to 38 of Q-Sepharose column; Panel (C): Lane P2, pool of fractions from Q-Sepharose column; Lane 65–79, fractions 65 to 79 of Sephacryl S-200 gel filtration column. Filled arrows indicate the position of B6Fabenv12CS protein and hollow arrows of free B6LC. Agglutination activity of the fraction before addition of 9E10 (A_b_) and after addition of 9E10 (A_a_) are shown in boxes. '0' indicates no visible agglutination. The number indicates maximum dilution of sample that gave visible agglutination.

After renaturation of B6LC and B6Fdenv12CS for assembly of B6Fabenv12CS, the renatured material was dialysed and pH of the protein solution was adjusted to 5.0, which led to some precipitation. On analysis by SDS-PAGE, the precipitate was found to contain mainly high molecular weight aggregates (data not shown). The precipitate was removed by centrifugation and filtration. The clear protein solution was loaded on SP-Sepharose column and bound proteins were eluted with a linear gradient of NaCl. Each fraction was analysed by SDS-PAGE and for RBC agglutination. B6Fabenv12CS eluted between 350 to 700 mM gradient of NaCl. SDS-PAGE analysis of peak fractions showed that fractions 25–37 contained a 58 kDa band corresponding to B6Fabenv12CS protein (Fig. 3A). In agglutination assay, fractions 35–38 showed agglutination before addition of 9E10 antibody indicating that these fractions contained aggregates. After addition of 9E10, strong agglutination was also observed in fractions 25–34. Fractions 26–35 were pooled, neutralised to pH 8.5 with 1M Tris solution, treated with iodoacetamide and loaded on desalting column of Sephadex G-25 which removed NaCl and free iodoacetamide from the sample. The protein pool obtained from G-25 column was loaded on 20 ml Q-Sepharose column. The Q-Sepharose column was developed with a linear gradient of NaCl and B6Fabenv12CS eluted between 100 to 300 mM gradient of NaCl. SDS-PAGE of peak fractions showed that fractions 24–30 contained band corresponding to B6Fabenv12CS protein while fractions 32–38 contained small amount of monomeric Fab (58 kDa) but large amount of high molecular weight species (Fig. 3B). In agglutination assay, fractions 32–40 showed agglutination before addition of 9E10 antibody indicating the presence of aggregates. After addition of 9E10, agglutination was also observed in fractions 24–30. Fractions 24–30 were pooled and loaded on Sephacryl S-200 gel filtration column to separate contaminating aggregated Fab molecules. Fractions 66–79 showed band corresponding to B6Fabenv12CS protein on SDS-PAGE with purity of ~90% (Fig. 3C). Fractions 70–74 containing the peak were not loaded on the gel. In agglutination assay, fractions 63–65 showed agglutination before addition of 9E10 antibody indicating the presence of aggregates. After addition of 9E10, agglutination was observed in fractions 66–79. Fractions 67–78 were pooled and stored. 9.2 mg of B6Fabenv12CS protein (>90% purity) was obtained from 2.3 liter of renatured material.

B6Fabenv12SC was purified following an identical protocol and the elution profiles were also similar to that of B6Fabenv12CS. 12.3 mg of B6Fabenv12SC protein was obtained from 3.0 liter of renatured material. B6Fabenv12CC was purified from the dialysed material following procedures as mentioned above. However, the amount of monomeric Fab obtained was very less. Most of the LC and Fdenv12CC were present in free form indicating that they had not associated to form Fab. 200 μg of B6Fabenv12CC protein was obtained from 1.6 liter of renatured material.

### Characterization of purified B6 Fab and B6Fabenv fusion proteins

Immunoreactivity of various purified proteins was studied using two normal, seven HIV-1 positive, five HIV-2 positive and four HIV-1+2 positive serum samples following a quantitative microtitre plate agglutination assay. The identity of the serum samples was based on several immunoassays, which are able to discriminate between antibodies to HIV-1 and HIV-2. In microtitre plate assay, HIV-1 positive samples showed strong agglutination with B6Fabenv1C coated RBCs and no reactivity with B6Fabenv2C or B6Fabenv2S coated RBCs (Table 2). Similarly, HIV-2 positive samples reacted strongly with B6Fabenv2C coated RBCs and gave no reaction with B6Fabenv1C and B6Fabenv1S coated RBCs. Samples positive for both HIV-1 and HIV-2 showed agglutination with both the proteins. The serum samples could be diluted to very high degree indicating the sensitivity of detection. B6Fab coated RBCs did not show agglutination with any of the serum samples except for control MAb 9E10 indicating the high specificity of the reagents. B6Fabenv12SS reacted with 13 HIV positive serum samples. However, two HIV-1 positive serum samples (Cal21 and Cal22) did not show agglutination with this protein. These two samples also did not react with single antigen protein B6Fabenv1S but reacted strongly with B6Fabenv1C (Table 2) indicating that cysteine residues were important for sero-reactivity. Due to paucity of these two samples, the double antigen molecules could not be tested. Nevertheless, positive reaction with B6Fabenv1C suggests the likelihood of the double antigen molecules also showing positive reaction with these samples. The HIV-1+2 samples showed strong reaction with double antigen molecules suggesting that both env1S and env2S sequences were recognised by antibodies present in the serum samples.

Three of the HIV-1 positive samples (C-91, C-92, C-93) showed very weak reactivity with B6Fabenv12SS and B6Fabenv12SC but were strongly reactive to two other proteins, B6Fabenv12CS and B6Fabenv12CC carrying cysteine residues in env1 sequence. This reactivity was similar to that of single antigen construct, B6Fabenv1C. These results indicate that it is essential to have cysteine residues in env1 sequence. On the other hand, HIV-2 positive samples showed strong reactivity with B6Fabenv12SS and all other double constructs, similar to single antigen molecule B6Fabenv2S and B6Fabenv2C, indicating that presence of disulfide bond forming cysteine might not be essential for immunoreactivity of env2. One HIV-1+2 sample (288/97) which showed strong reactivity to B6Fabenv1C and weak reactivity to B6Fabenv2C was reactive to all double antigen constructs and showed additive reactivity. None of the double antigen molecules showed agglutination with the non-HIV samples indicating their high specificity.

## Discussion

The work described here clearly demonstrates that anti-human RBC Fab fusion proteins carrying sequences from both envelope gp41 of HIV-1 and envelope gp36 of HIV-2 in the same Fab molecule can be produced for use in haemagglutination based detection of antibodies to HIV in whole blood.

A large number of fusion proteins were designed for this study, all of which carried an anti-human RBC Fab protein fused to epitopes from both HIV-1 gp41 (env1) and HIV-2 gp36 (env2) in tandem. Initially double antigen construct carrying serine residues, in place of cysteine residues present in wild type envelope sequences, was constructed. This protein, named B6Fabenv12SS, contained Fab protein linked to env1S followed by a Gly-Ser linker and env2S with cmyc tag at the end. Evaluation of B6Fabenv124SS in comparison to single antigen constructs showed that the double antigen construct could simultaneously detect antibodies to both HIV-1 and HIV-2 in serum samples, with sensitivity comparable to that of single antigen construct containing serine in envelope (B6Fabenv1S and B6Fabenv2S). However, two HIV-1 positive samples, Cal21 and Cal22 were missed by these proteins but were detected by another single antigen construct carrying cysteine in envelope (B6Fabenv1C). With several samples, single antigen fusion proteins carrying native envelope sequence of HIV-1 gp41 (with cysteine residues) were more sensitive than those carrying serine residues in envelope sequence. Thus, it was important to make double constructs with wild type sequences of HIV-1 gp41 and HIV-2 gp36 (with cysteine residues) for improved sensitivity. At the same time it was also noted that cysteine residues in envelope sequences in close vicinity to each other and to the associating LC and Fd chain cysteines' could interfere with correct interchain and intrachain disulfide formation.

Therefore, B6Fabenv12SS was converted into three types of molecules containing different combination of envelope sequences: (i) Fabenv12CS, containing sequence of native gp41 of HIV-1 (env1C) followed by linker and sequence of gp36 of HIV-2 having serine residues in place of cysteine residues (env2S); (ii) Fabenv12SC, containing sequence of gp41 of HIV-1 having serine residues in place of cysteine residues (env1S) followed by linker and sequence of native gp36 of HIV-2 (env2C), and (iii) Fabenv12CC, containing sequence of native gp41 of HIV-1 (env1C) followed by linker and sequence of native gp36 of HIV-2 (env2C).

The double antigen fusion proteins of B6 were purified using a series of column chromatography procedures. On purification, B6Fabenv12CS and B6Fabenv12SC gave good yields that were comparable to yields of B6Fabenv12SS and single antigen constructs of B6, indicating that all the three domains, namely, Fab, env1 and env2 were folding properly (Fig. 2). However, the yield of B6Fabenv12CC was 20 times less compared to that of other proteins, indicating that the molecule was not folding properly. Detailed analysis showed that the association of Fd and LC was defective in B6Fabenv12CC. It was thought that the spacer between the envelope sequences comprising of glycine and serine residues may be very flexible, thus bringing the cysteine residues in envelope sequences close to each other and in close proximity to LC and Fd causing interference in correct folding of various domains and assembly of LC and Fd to form Fab. The flexible Gly-Ser linker was replaced by other, less flexible linkers, comprising of proline residues. However, there was no improvement in the assembly of LC and Fd chains and in overall yield of proteins (data not shown).

The double antigen proteins were tested for their efficacy in detecting anti-HIV antibodies in human serum samples in comparison to single antigen proteins. All the double antigen proteins showed good reactivity to HIV positive samples with no cross-reactivity to non-HIV samples. B6Fabenv12SS showed weaker reactivity to samples, especially to HIV-1 infected samples, as compared to other proteins containing native envelope sequences (Table 2). B6Fabenv12CS and B6Fabenv12SC showed very interesting reactivity pattern to samples having only anti-HIV-1 antibodies. B6Fabenv12CS showed good reactivity to all the samples, comparable to that of B6Fabenv1C. However, B6Fabenv12SC showed weaker reactivity similar to that shown by B6Fabenv1S, emphasizing the importance of native cysteine residues in the envelope sequence for immunoreactivity. All proteins showed good reactivity to HIV-2 samples. Also, HIV-1+2 samples (288/97 and PRZ 202-12) showed additive effect, with reactivity in double antigen proteins being more than in single antigen proteins (Table 2). This indicates that both the envelope antigens are being recognised equally well when present in a single protein, with no difference in sensitivity and specificity compared to single antigen fusion proteins.

## Conclusion

The paper describes successful designing and production of new, bifunctional, recombinant molecules for detection of anti-HIV antibodies in blood of HIV-infected individuals. The results described above strengthen the hypothesis that for higher sensitivity of detection, it is essential to have native sequences of HIV-1 gp41 and HIV-2 gp36. B6Fabenv12CC, therefore is the ideal molecule but its yields are very low (Fig. 2). However, from this study, B6Fabenv12CS emerges as a potential molecule for simultaneous detection of antibodies to both HIV-1 and HIV-2. In addition to giving good yield on purification, B6Fabenv12CS showed good reactivity to all HIV-1 samples (Fig. 2). Also, it showed good reactivity to all HIV-2 samples inspite of having env2S sequence. This may be due to high titre of antibodies in all the HIV-2 samples tested in this study. It is difficult to speculate the reactivity of this protein to low titre HIV-2 samples. It is important to note that although HIV-2 infection is prevalent in India, most of the cases have dual infection with very few cases having only HIV-2 infection. Therefore, B6Fabenv12CS should show good reactivity to most samples.

The results clearly suggest B6Fabenv12CS as the molecule of choice for simultaneous detection of antibodies to both HIV-1 and HIV-2, however, it needs to be evaluated for reactivity to more samples before large-scale use. The technology described in this paper can also be used for development of similar chimeric molecules for simultaneous, multiplex detection of various infections, such as Hepatitis B, Hepatitis C and Syphilis, which are relevant to blood transfusion.

## Methods

### Materials

MAb B6 was isolated by conventional hybridoma technology in our laboratory using spleen cells from mice immunised with O RhD negative human RBCs. The universal reactivity of this antibody was established by screening more than 1000 random blood samples. The DNA encoding LC and Fd of B6 were cloned by PCR based methods using consensus primers annealing to framework regions of B6 (our unpublished data). Q-Sepharose fast flow, SP-Sepharose fast flow, Sephacryl S-200 (high resolution), Sephadex G-25 (medium) and chromatography columns were purchased from GE Healthcare. Human serum samples were obtained from Post Graduate Institute of Medical Education and Research (PGI), Chandigarh, India, Christian medical college (CMC), Vellore, India and Centre for Disease Control (CDC), Atlanta, USA or purchased from Boston Biomedica Inc. (BBI), USA. These were previously collected serum samples that had been characterised for HIV infection using immunoassays. For this study, they were provided as coded samples and identity of the donor was not disclosed.

### Expression vector

A high copy number T7 promoter based expression vector, pVCCD41140 [6] was used for cloning antibody sequences. The vector utilizes *E. coli *host BL21(λDE3) that carries λ lysogen encoding T7 RNA polymerase gene under the control of *lacUV5 *promoter inducible by IPTG [8]. In this vector, DNA encoding antibody is cloned as *Nhe *I-*Mlu *I fragment. The vector carries T7 transcription terminator and origin of replication of filamentous phage for making single stranded DNA. Different plasmids were assembled using standard cloning techniques and their sequence was confirmed by DNA sequencing (ABI Model 377). The characteristics of each plasmid are depicted in Fig. 1.

pVCB6Fd contains DNA sequence encoding Fd of anti-RBC monoclonal antibody, B6, as *Nhe *I-*Mlu *I fragment followed by sequence for a 10 amino acid tag, cmyc. pVCB6LC contains DNA sequence encoding light chain of B6 (B6LC) as *Nhe *I-*Mlu *I fragment with a stop codon preceding *Mlu *I site.

Oligonucleotide adaptors encoding immunodominant epitopes of gp41 of HIV-1 (amino acid 590 to 620 of HIV-1 envelope [9] with cysteine 605 and cysteine 611 replaced by serine residues) and gp36 of HIV-2 (amino acid 581 to 611 of HIV-2 envelope [10] with cysteine 597 and cysteine 603 replaced by serine residues) were assembled from synthetic oligonucleotides and cloned into pVCB6Fd resulting in formation of pVCB6Fdenv1S and pVCB6Fdenv2S, respectively. The construction of plasmids, pVCB6Fdenv1C and pVCB6Fdenv2C which contain sequence for env1 and env2 with cysteine residues was carried out using similar strategy.

### Construction of various plasmids to express Fd fused with both HIV-1 and HIV-2 antigens

DNA encoding env1 was amplified from pVCB6Fdenv1S by PCR and cloned in vector pVCB6Fdenv2S and the resultant recombinant was named pVCB6Fdenv12SS.

Oligonucleotides env14CC (5'-ACAGCAGTGGTGCATATCAGCTTTCCTGAGCAACCCCAAATC-3') annealing to env1S sequence and containing anticodon sequence for the cysteine residues (underlined) and env24CC (5'-ACCGTGGTGTGGCAGACCTGGCGGAATGCGCATC CCCAGCTG-3') annealing to env2S sequence and containing anticodon sequence for the cysteine residues (underlined) were used simultaneously for mutagenesis using uracil containing ssDNA template of pVCB6Fdenv12SS and following Kunkel's method of mutagenesis [11]. The transformants were analyzed by restriction enzyme digestion and the sequence was confirmed by DNA sequencing. Mutants carrying change of serine to cysteine in env1 only were named pVCB6Fdenv12CS and those with mutation of serine to cysteine in env2 only were named pVCB6Fdenv12SC. Double mutants carrying cysteine residues in both env1 and env2 were named pVCB6Fdenv12CC.

### Expression and isolation of inclusion bodies

BL21 (λDE3) cells were transformed with various plasmids and grown on LB plates containing ampicillin for 16 hours at 30°C. Transformed cells from 6 plates (approximately 2000 colonies each) were scrapped and inoculated in 1 litre superbroth containing ampicillin, and grown at 30°C with vigorous shaking. At OD_600 nm _of 2.5–3.0, the culture was induced with IPTG (final concentration, 0.25 mM) and grown at 30°C. After 2 hours of induction, the culture was chilled over ice. Cells were harvested by centrifugation at 4000 × g (GSA rotor, Sorvall RC5C) for 10 min at 4°C.

Inclusion bodies were extracted by lysing the cells with Triton X-100 and following a protocol as described earlier [6]. The pellet containing inclusion bodies in Oak ridge tubes was stored at -70°C till further use. The inclusion bodies prepared were analysed on 0.1% SDS-12.5% PAG.

### Assembly of Fab molecules

Inclusion Bodies isolated from one litre culture each of B6Fd or B6Fdenv fusion protein and B6LC were solubilised in 10 ml solubilisation buffer (0.1 M Tris.HCl, pH 8.0, 2 mM EDTA, 6 M Guanidine HCl. Total protein of solubilised Fd/Fd fusion protein and LC was estimated by Bradford method and the final protein concentration was adjusted to 10 mg/ml. Equimolar amounts of B6LC and B6Fd or B6Fdenv fusion proteins were taken and Dithioerythritol (DTE) was added to a final conc. of 10 mg/ml and incubated at room temperature for 2 hours. After this, LC and Fd solutions were mixed and centrifuged at 93,000 × g, for 30 minutes. The supernatant was collected and diluted 100 fold in cold renaturation buffer (0.1 M Tris.HCl, pH 8.0, 2 mM EDTA, 0.5 M Arginine HCl, 0.9 mM oxidised Glutathione). This material was kept undisturbed at 10°C for 70 hours for renaturation.

The renatured material was dialysed against three volumes of 20 mM Tris, pH 8.0, containing 100 mM Urea. Dialysis was done for 72 hours with 4 buffer changes. The final dialysis was done in buffer containing 50 mM Urea. The dialysed material was filtered through 2.3 μm and 0.4 μm filters and then used to purify renatured, monomeric, Fab/Fabenv fusion proteins.

### Purification of Fab proteins

After renaturation, the assembled monomeric Fab fusion protein was purified using a series of column chromatographic steps. These steps were common for various fusion proteins. Therefore, purification of only B6Fabenv12CS (made by assembling B6LC and B6Fdenv12CS) is described below in detail.

The pH of the dialysed and filtered solution containing Fab fusion protein was adjusted to ~5.0 with 5% acetic acid and the sample were kept undisturbed at 4°C for 12 hours. After centrifugation at 23,500 × g, the supernatant was filtered through 0.4 μm filter. Using FPLC, the filtrate (3.5 liter) was applied at a flow rate of 12 ml/min on a 50 ml SP-Sepharose column (XK 50/20) pre-equilibrated with 20 mM acetate buffer, pH 5.0 (Buffer A). All the chromatography steps were carried out at 2–8°C. The column was washed with 100 ml of Buffer A containing 100 mM NaCl at a flow rate of 8 ml/min and the protein was eluted with a 250 ml linear gradient of NaCl (100–600 mM) in Buffer A. The elution was monitored at 280 nm and fractions were collected. The fractions (12 ml each) were neutralized immediately with 120 μl 1 M Tris solution. The fractions containing the desired protein (analysed by SDS-PAGE under non-reducing conditions and by RBC agglutination assay) were pooled and pH was adjusted to ~8.5 by adding 1M Tris solution. Iodoacetamide was added to a final concentration of 50 mM and the protein solution was kept in a water bath at 25°C for 15 minutes. The iodoacetamide treated sample was applied on a 325 ml Sephadex G-25 column (XK 26/70) pre-equilibrated with 20 mM Tris Buffer, pH 8.5 (Buffer C). After loading, the protein was eluted in Buffer C at a flow rate of 5 ml/min with monitoring at 280 nm and collection of 12 ml fractions. Based on absorbance values, fractions containing Fab were pooled. The pool was loaded on a 20 ml Q-Sepharose column pre-equilibrated with Buffer C. The column was washed with 40 ml of Buffer C at a flow rate of 4 ml/min and the protein was eluted with a 120 ml linear gradient of NaCl (0–350 mM) in Buffer C. The elution was monitored at 280 nm and 4 ml fractions were collected. The fractions were analysed by SDS-PAGE under non-reducing conditions and by agglutination assay. The fractions containing desired protein were pooled and the pool was loaded on 500 ml Sephacryl S-200 gel filtration column (XK 26/100) pre-equilibrated with 20 mM phosphate buffer, pH 7.0 containing 150 mM NaCl (PBS). The column was developed with PBS at a flow rate of 1 ml/min. The elution was monitored at 280 nm and 4 ml fractions were collected and analysed. The fractions containing active, monomeric Fab fusion protein were pooled.

### Agglutination assay of purification samples

60 μl of column fraction or an appropriate dilution (including sample before column loading and flow through obtained during loading) was taken in a 96-well round-bottom microtitre plate (Wells 1–12). Two-fold serial dilution were made in PBS-BSA (PBS containing 0.2% BSA) (Wells A-H). To each well, 30 μl of 2% human RBC suspension (washed RBC pellet reconstituted v/v to 2% in PBS-BSA) was added. After mixing, the plate was incubated at 37°C for 1 hour. Agglutination was read visually and the maximum dilution of sample that gave agglutination (end point) was recorded. The plate was centrifuged at 800 × g at RT for 5 minutes. The supernatant was discarded by inversion and the pellet was suspended in remaining buffer by vortexing. 60 μl of anti-cmyc MAb (1:1000 dilution of 9E10 ascitic fluid in PBS-BSA) was added per well. After mixing, the plate was incubated at 37°C for 1 hour. Agglutination was read as above. Samples, which gave agglutination only after addition of 9E10 antibody, contain active monomeric Fab/Fab fusion protein.

### Quantitative plate agglutination assay to detect anti-HIV antibodies in serum

Human RBCs of O RhD negative blood were washed twice with PBS-BSA. To 2% RBC suspension, purified B6Fab/B6Fabenv fusion protein was added to a final concentration of 1 μg/ml. The contents were mixed by tapping and inversion and incubated at 37°C for one hour with intermittent mixing, to produce coated RBC suspension. Separately, two-fold serial dilution of human sera (starting from initial dilution of 100-fold) were made in PBS-BSA (Wells A-H). To each well containing serum dilution, 30 μl of coated RBC suspension was added. After mixing, the plate was incubated at 37°C for 1 hour and agglutination was recorded.

## Abbreviations

cmyc, decapeptide recognised by monoclonal antibody 9E10; env1, 31 amino acids (590–620) of HIV-1 envelope glycoprotein (gp41); env2, 31 amino acids (581–611) of HIV-2 envelope glycoprotein (gp36); *E. coli*, *Escherichia coli*; Fd, fragment consisting of variable domain (V_H_) and the first constant domain (C_H_1) of the heavy chain of antibody; Fab, antigen binding fragment; Fdenv, Fd fragment carrying 31 amino acid peptide from envelope glycoprotein of HIV-1 or HIV-2; Fabenv, Fab fusion protein carrying 31 amino acids from envelope glycoprotein of HIV-1 or HIV-2; IgG, immunoglobulin type G; IPTG, isopropyl-β-D-thiogalactoside; LC, Light-chain of an antibody; MAb, monoclonal antibody; PBS, phosphate buffered saline (20 mM phosphate buffer, pH 7.2 containing 145 mM NaCl); RT, room temperature; RBC, human red blood cell.

## Competing interests

The molecules described in this paper have commercial potential and a patent application for the same is being processed.

## Authors' contributions

Both the authors have been involved in all aspects of the described work.

## Figures and Tables

**Table 1:** Alignment of amino acid sequence of envelope region of various single and double antigen fusion proteins.

Protein	**AMINO ACID SEQUENCES OF HIV-1 and HIV-2 ANTIGENS FUSED AT THE C-TERMINUS OF Fd**
	Fd------- -------ENV1S/C-------------LINKER L-----------------ENV2S/C----------------cmyc

Fd	NASG
Fdenv1S	NASGVERYLKDQQLLGIWG**S**SGKLI**S**TTAVPWNAS----------------------------------------------EQKLISEEDLN
Fdenv1C	NASGVERYLKDQQLLGIWG**C**SGKLI**C**TTAVPWNAS----------------------------------------------EQKLISEEDLN
Fdenv2S	NASG------------------------------------------ AIEKYLQDQARLNSWG**S**AFRQV**S**HTTVPWVNGASEQKLISEEDLN
Fdenv2C	NASG------------------------------------------ AIEKYLQDQARLNSWG**C**AFRQV**C**HTTVPWVNGASEQKLISEEDLN
Fdenv12SS	NASGVERYLKDQQLLGIWG**S**SGKLI**S**TTAVPWSN**ASGGGSGGGASG**AIEKYLQDQARLNSWG**S**AFRQV**S**HTTVPWVNGASEQKLISEEDLN
Fdenv12CC	NASGVERYLKDQQLLGIWG**C**SGKLI**C**TTAVPWSN**ASGGGSGGGASG**AIEKYLQDQARLNSWG**C**AFRQV**C**HTTVPWVNGASEQKLISEEDLN
Fdenv12CS	NASGVERYLKDQQLLGIWG**C**SGKLI**C**TTAVPWSN**ASGGGSGGGASG**AIEKYLQDQARLNSWG**S**AFRQV**S**HTTVPWVNGASEQKLISEEDLN
Fdenv12SC	NASGVERYLKDQQLLGIWG**S**SGKLI**S**TTAVPWSN**ASGGGSGGGASG**AIEKYLQDQARLNSWG**C**AFRQV**C**HTTVPWVNGASEQKLISEEDLN

Amino acids are shown in single letter code with Serine (S) and Cysteine (C) in bold letters. The linker is shown in bold letters. Various proteins are shown diagrammatically in Fig. 2.

**Table 2:** Reactivity of various recombinant B6Fab fusion proteins to human serum samples

**S.No**	**SERUM SAMPLE**	**SOURCE OF SAMPLE**	**IDENTITY**	**DILUTION OF SERUM GIVING END POINT OF AGGLUTINATION**								
				
				B6Fab	B6Fab	B6Fab	B6Fab	B6Fab	B6Fab	B6Fab	B6Fab	B6Fab
				env1S	env1C	env2S	env2C	env12SS	env12SC	env12CS	env12CC	
1.	Cal 21	Calcutta	HIV-1	0	1600	nd	nd	0	nd	nd	nd	0
2.	Cal 22	Calcutta	HIV-1	0	1600	nd	nd	0	nd	nd	nd	0
**3.**	**C-91**	**Vellore**	**HIV-1**	**nd**	**12800**	**nd**	**0**	**1600**	**6400**	**12800**	**12800**	**0**
**4.**	**C-92**	**Vellore**	**HIV-1**	**nd**	**12800**	**nd**	**0**	**800**	**1600**	**12800**	**12800**	**0**
**5.**	**C-93**	**Vellore**	**HIV-1**	**nd**	**51200**	**nd**	**0**	**0**	**12800**	**51200**	**51200**	**0**
6.	C-95	Vellore	HIV-1	nd	>25600	nd	0	>25600	>25600	>25600	>25600	0
7.	PRZ202-07	BBI, USA	HIV-1	nd	25600	nd	0	3200	6400	>25600	25600	0
8.	266/97	Chandigarh	HIV-1+2	12800	>25600	1600	3200	25600	>25600	>25600	>25600	0
**9.**	**288/97**	**Chandigarh**	**HIV-1+2**	**3200**	**25600**	**800**	**3200**	**6400**	**6400**	**>25600**	**25600**	**0**
10.	C-74	Chandigarh	HIV-1+2	nd	>25600	nd	25600	25600	>25600	>25600	>25600	0
11.	**PRZ202-12**	**BBI, USA**	**HIV-1+2**	**nd**	**1600**	**nd**	**25600**	**6400**	**25600**	**12800**	**25600**	**0**
12.	575/97	Chandigarh	HIV-2	0	0	>25600	>25600	25600	>25600	>25600	>25600	0
13.	PRZ202-01	BBI, USA	HIV-2	nd	0	nd	>25600	>25600	>25600	>25600	>25600	0
14.	PRZ202-03	BBI, USA	HIV-2	nd	0	nd	12800	6400	12800	12800	12800	0
15.	PRZ202-05	BBI, USA	HIV-2	nd	0	nd	>25600	>25600	>25600	>25600	>25600	0
16.	PRZ202-09	BBI, USA	HIV-2	nd	0	nd	6400	3200	6400	6400	6400	0
17.	871	Chandigarh	Negative	0	0	0	0	0	0	0	0	0
18.	872	Chandigarh	Negative	0	0	0	0	0	0	0	0	0
19.	9E10	MAb	Control	25600	25600	12800	25600	12800	>25600	>25600	>25600	25600

Identity of samples is based on data provided by sample supplier as indicated in source of sample. Description of various fusion proteins is given in Fig. 2.

nd-not done

## References

[B1] (2001). AIDS epidemic update.

[B2] (1998). Update: HIV counseling and testing using rapid tests--United States, 1995. MMWR Morb Mortal Wkly Rep.

[B3] Kassler WJ, Dillon BA, Haley C, Jones WK, Goldman A (1997). On-site, rapid HIV testing with same-day results and counseling. Aids.

[B4] Gupta A, Chaudhary VK (2003). Whole-blood agglutination assay for on-site detection of human immunodeficiency virus infection. J Clin Microbiol.

[B5] Rudolph R, Buchner J. (1991). Renaturation, Purification and Characterization of Recombinant Fab-Fragments produced in Escherichia coli. Bio/Technology.

[B6] Gupta A, Gupta S, Chaudhary VK (2001). Recombinant fusion proteins for haemagglutination-based rapid detection of antibodies to HIV in whole blood. J Immunol Methods.

[B7] Gnann JWJ, Nelson JA, Oldstone MB (1987). Fine mapping of an immunodominant domain in the transmembrane glycoprotein of human immunodeficiency virus. J Virol.

[B8] Studier FW, Rosenberg AH, Dunn JJ, Dubendorff JW (1990). Use of T7 RNA polymerase to direct expression of cloned genes. Methods Enzymol.

[B9] Ratner L, Haseltine W, Patarca R, Livak KJ, Starcich B, Josephs SF, Doran ER, Rafalski JA, Whitehorn EA, Baumeister K (1985). Complete nucleotide sequence of the AIDS virus, HTLV-III. Nature.

[B10] Clavel F, Guetard D, Brun-Vezinet F, Chamaret S, Rey MA, Santos-Ferreira MO, Laurent AG, Dauguet C, Katlama C, Rouzioux C (1986). Isolation of a new human retrovirus from West African patients with AIDS. Science.

[B11] Kunkel TA (1985). Rapid and efficient site-specific mutagenesis without phenotypic selection. Proc Natl Acad Sci U S A.

